# Supplementary material for: MetaRibo-Seq measures translation in microbiomes
Source: Nat Commun. 2020 Jun 29;11:3268. doi: 10.1038/s41467-020-17081-z (PMC7324362; doi:10.1038/s41467-020-17081-z)
Supplement: Supplementary file 10 — Supplementary Data 7 [file 41467_2020_17081_MOESM10_ESM.zip › File2/Confidence_High_Taxonomy/34843_out.krona.html]

Javascript must be enabled to view this page.

members
magnitude
magnitudeUnassigned
count
unassigned
taxon
rank

34843\_out

2

superkingdom
2
2

1239
2
phylum

186801
2
class

order
2
186802

186803
2
family


SRS015578\_contig\_number\_20648
39491
1
species

841
1
genus


SRS042628\_contig\_number\_contig-100\_1469.201125
2293129
1
species
